# Valedictory Address to the Graduates of Medical Department of Lind University, for the Session of 1860-61

**Published:** 1861-03

**Authors:** M. K. Taylor

**Affiliations:** Professor of Pathology and Public Hygiene


*Valedictory Address to the Graduates of Medical Department of Lind University, for the Session of* 1860-61. By M. K. Taylor, M. D., *Professor of Pathology and Public Hygiene.*

Gentlemen.—We have assembled, probably, for the last time, in the relation of teachers and pupils. The prescribed course of instruction has terminated. Your proficiency has been endorsed, and with the seal of this University you will soon be wending your way to your respective homes, to receive the congratulations of friends, and to enter, we trust, upon a more extended sphere of usefulness.

The anxieties, aspirations, desires and possibly rivalries of studentship are passed, and the hopes and fears of a final examination are quieted in the reception of the testimonials this night conferred, and in this more public acknowledgement of your industry and worth. And while we thus recognise your acquirements in medical science, and assure the public so far as we may, by these outward acts, that you are justly entitled to its confidence and support, we take occasion to express our own gratification in knowing that you have made every reasonable effort to qualify yourselves thoroughly for the responsibilities you propose to assume. Indeed, I may go farther than this, and declare our belief that few students in this country will enter the ranks of the medical profession better prepared to discharge its obligations than yourselves. I say not this with the view of tickling your vanity or pride, nor as an unmeaning compliment to sweeten the hour of parting, but to express more fully our firm conviction that the advantages growing out of the plan of instruction herein adopted, together with your own ardent desires to improve them, have been conducive in a greater degree to this end. Feeling thus, therefore, it is with more than an ordinary amount of pleasure that I welcome you into the ranks of one of the most responsible, and when rightly viewed, one of the most ennobling pro fessions of civilized life. I cheerfully extend to you the right hand of fellowship and meet each of you with a cordial greeting, and that fraternity of sentiment which you have a right to expect. Rest assured of the interest we shall feel in your future welfare, and of the pleasure it will afford us to hear of your success in life, and to know hereafter that nothing shall have transpired causing you to regret this choice of a vocation. We hope, too, that the same zealous spirit of inquiry and observation which we have witnessed during your pupilage will continue beyond the shadows of the institution you are now about to leave, and remain an abiding influence throughout you professional career, and at last meet with that reward in honor, position, and profit, to which true merit may justly lay claim. I would also with this assumption of a new position have you take enlarged views of what should be the great aims of life in the promotion of the welfare of your fellow creatures; and bear in mind that you have a two-fold character to sustain before the world, in your personal and professional relations, the dignity of which can be best upheld and vindicated by exemplification in your own conduct. Your duties in this respect, therefore will be the subject of our special consideration.

In entering upon your professional duties, and having a proper regard for a faithful discharge of them, you must expect at the outset, that every virtue of the human heart, and the highest qualities of the understanding, will, more or less, be called into requisition.

For the exercise of a sound discretion, a good judgement, a desire to please without a forfeiture of self respect, a high sense of honor, an unbending integrity, kindness, sympathy, gentleness, without loss of efficiency, a liberal allowance for the peculiarities of some, and the weakness of the infirm, you will continually find drafts made upon you, and it is to be hoped that none of these demands will ever become subjects of protest.

But beyond these appeals to, and demands upon the sterling virtues you may possess, there is the sense of responsibility standing as an ever present monitor in the minds of those who feel they have an account to render to their own consciences as well as to their Maker, for the short comings of this world. Life, health, and all its blessings, the richest pleasure of earth, the support of declining years, and all the hopes that brighten the pathway of age to the tomb, are often intrusted to the physician's care. The great and powerful, rich and poor, president and people, alike bow to our professional shrine, and seek at the hands of its votaries relief for their multiform mental and physical infirmities. This sense of responsibility is to be cherished, and the moral obligations for the proper performance of your duties, should ever be subjects of your serious consideration. But not only this, for thinking without action has but little merit in it, we enjoin upon you to be diligent in the acquisition of more knowledge as well by your own patient investigation and studies, as in keeping pace with the labors and advancement of others. To be a student, therefore, is a necessity through life, otherwise these responsibilities cannot be honestly discharged.

You must by this time be well aware that the field before you for the exercise of your mental faculties is an unlimited one. Indeed it may be said to reach beyond any single human capacity, for medical science is the sum of all others, and has for its basis all that we know of the mental, moral and physical relations of mankind to the world.

Its vast extent, to some, may be a source of discouragement. Yet boundless as it may appear, and unpromising as it may seem to one desirous of grasping a given amount of knowledge only, and appropriating it solely to selfish purposes, medical science has allurements to the true student, which are unequalled by any other, and which lead him to the loftiest conceptions of the subtleties of life, and all its attendant intricacies and surroundings.

It will give the close investigator, a catholicity of spirit, and teach him to hold up before the world his profession unspotted by selfishness—uncontaminated by dishonor or meanness, to cherish a fraternal regard for his professional bretheren, and at all times to feel an abiding faith in its ultimate triumph over any and all opposing, so called, systems of medicine, come they from whatever source they will.

These are but a few of the encouragements that I would offer as a stimulation to unceasing devotion to your profession, both in respect to its literature and science, as well as their practical application in the removal or alleviation of disease. They pertain mainly to yourselves individually, and bear more forcibly upon the inner consciousness of one seeking at all times to be true to himself first, and then to those who in their hour of suffering may require his aid.

But while, for your own sake I would urge the importance of an assiduous devotion to the more intellectual departments of your profession, I am by no means unmindful of the obstacles which will arise from business relations, the discouragements growing out of a want of appreciation of one's labors by the public—for a time, perhaps possibly forever; and the tempting allurements offered by the credulity of men, the preying upon which may lead to affluence and high social position. 'Tis true, these blighting influences have done much to weigh down and reduce to sluggards and routinists, many of those, whose aspirations in early life led them in the direction of better things, and even have driven many from the paths of professional rectitude. Still I trust that none of these will, even for a moment, deter you from obeying the behests of your medical obligations, and of meeting them with the stronger weapons of indomitable perseverance. Closely associated with these obstacles to your progress, however, and that which operates with the greatest force upon all members, old and young, of our profession, is the ignorance of a large majority of the public, of what constitutes the basis of the principles and practice of medicine. With these, medicine is a matter of belief only, and not of any positive knowledge. Hence the most absurd crudities and superstitions find as ready a lodgment in their minds as the more potent facts of medical science. Indeed it would almost seem that the rapid advancements in all departments of physical knowledge, and the ready promulgation of the same, through a teeming press, has served to unsettle the minds of many in the application of the plainest common sense rules, involved in the ordinary transactions of life, to the treatment of their ailments, and engendered a morbid desire for something new and startling, and totally distinct from anything heretofore considered a fixed principle.

Novelty in such cases, usurps the place of a conservative stability—the new the place of the old—and under the deceitful and high sounding name of progress, with the zeal of a Mohamedan, they unceasingly proclaim their crude notions of the most abstract sciences, and at the same time dispaly their unblushing ignorance of the basis upon which any single one is founded. Neither does it matter where these notions originate. They are alike, ready to accept as an established truth every " pathy and ism " emanating from Boston or Germany, as well as the less pretentious superstitions, and wierd fancies of a Pattowatamie Squaw; nor, is it alone among the less cultivated and uninformed, that you will be likely to meet these. Men of intelligence in other matters, are not free from them, and you will often find the crude theories of an Egyptian age, clothed in the habiliments of a higher modern civilization. It will seem strange to you that these things exist, and you may be loth to accord that consideration to the opinions of men on medical subjects, which, upon every other, would give you the greatest pleasure. So, too, at times, you may feel nettled by a total disregard of what you concieve, and probably know to be for the best interests of those seeking your advice, and the straying away after their erratic fancies, those whom you have heretofore considered your most reliable friends. Nor need this be a matter of surprise. For, if you will call to mind the fact, that nearly all the physical sciences have been raised to their present gigantic proportions, since the commencement of the present century; that anything like a positive knowledge of the organic changes taking place in the human system, either in health or disease, dates back to scarcely half that time, that the common mind must necessarily, in most instances, be years behind the pioneers of scientific advancement, even so far as the general principles are involved, and that more especially the intrinsic difficulties attending investigations of a science so complicated as medicine, and which require years of patient industry to catch a gleam of its great truths; we may well excuse errors of opinion in the less informed, and treat their foibles so far as consistent with self respect, with a spirit of forbearance. Since, in the language of another, it is a well known fact, that whilst the public can judge with a tolerable degree of correctness, of merit in every other profession, it is wholly incapable of forming a proper estimate of ability in medicine. The structures of the human body, the functions they perform, the derangements to which they are liable, are to mankind in general, completely unknown. All that your patients will concern themselves with, are results, but so little are they familiar with the modes by which these are obtained, so little do they know of the operation of nature as distinguished from art, that they are especially liable to the most erroneous conclusions. Coupling these to the greedy avidity with which most persons seize upon all knowledge of this kind, without the ability to discriminate between the good and bad, or the source whence it emanates, it is not at all surprising that they should be unstable in their opinions, and drift in the direction of every point of the compass save the right one. For this reason, too, unprincipled persons from time immemorial, have practiced on public credulity, and some specious but shallow theory, some vaunted nostrum, some peculiar accomplishment, or some singularity of manner have each in turn been made the means of imposition, have each found favor in the estimation of large and even respectable classes in many communities, and for the time overridden the greater truths and practice of a rational science. These things exist, and we may speak of them as they are, and meet them like men. They become sources of annoyance to those who have a just conception of the highest aims of our art, and seek to be governed by a conscientious regard for the performance of the functions of a true physician. In the weaker, too, as well as in the indolent, they give rise to repinings and puling reflections on the want of appreciation and confidence in their ability—overwhelm them with discouragements—and reduce the practice of medicine, by them, to the meanest drudgery of their lives.

But to him who stands shielded by the panoply of a high scientific attainment, who has courage and an unwavering perseverence, to surmount all obstacles in the way of his progress—industrious habit of thought—and complete devotion to the welfare of his profession, they only aside from the annoyance of contact, serve as incentives to the more vigorous prosecution of what should be the great aim of his life—commanding, thereby, the confidence and respect of those with whom he associates, and making them feel that the position of an enlightened medical man, is as much above that of vaunting pretenders, as the blue vault of heaven is above the unfathomable depths of the earth beneath. Your duties in respect to correcting these evils are manifold and cannot be specified here. Suffice it to say, however, that a habit of idleness does not belong to them. On the contrary, you have to study, to think, to labor, as you have never done before. But while I urge the importance of an assiduous attention to the perfection of yourselves, as far as can be done in your medical studies, I would also call to mind your relations to the public, as conservators of its general welfare, and your obligations to correct these evils, so far as you may, by your personal influence and scientific acquirements. This should be done by lending a liberal support to every reasonable effort for the education of the masses. Let the crude theories of men be met by the abundant and overwhelming facts at your command. The weapons of a true science are all powerful upon the understanding, if you have the skill to handle them well. Ignorance and prejudice may for a time baffle you, but remove these by a liberal education, and the work is then easy with the aid of time. I do not mean, however, simply a general education, as is commonly understood in the popular use of the term, but in addition to that, the introduction into all our institutions of learning, of such a course of study in the elementary branches of medicine, as shall give a clear insight into some of its great fundamental principles. It has been said: "The proper study of mankind is man," and I suppose few will attempt to controvert the abstract proposition. Still, in the scheme for the instruction of the rising generation, but little advancement has been made towards the attainment of this end. All the efforts thus far are of the most superficial character. Speaking for myself, then, I may express the belief that the establishment of professorships of Anatomy and Physiology in every college and school, of such importance as to warrant it, when aided by a spirit of liberality on the part of the members of our profession, will do more than any other plan yet presented for our consideration.

I have not time to discuss the subject farther here, nor is it appropriate that I should, but this I may say, before dismissing it, that I trust you will ever be found prompt supporters of every effort in this direction, on account of the reactionary benefits accruing to yourselves, should no higher considerations move you to the performance of them.

So, in matters of public policy, of religion, of morality, law and order, and public sanitary relations, you may do much towards winning the confidence of your respective communities by a disinterested benevolence, and thereby control or guide them when under the specious influences of flippant so-called advocates of progression. Look not for immediate reward at the hands of others, for all you may do in matters of this kind, for it may never come. The greater consideration of having faithfully carried out the behests of an enlightened understanding of the duties of a modern physician should be sufficient. No groveling idea that all you do is to redound to selfish purposes should be entertained, for these are the lowest motives that can actuate men, and when carried out, have a tendency to narrow the mind, to make bigots, and render one odious in the extreme to nearly all classes of community. You live in an age when medical science, as well as all others, is making rapid strides in the direction of a true progress towards the greatest ulterior results. The whole medical world is busy with its investigations of the laws of life, in health and disease, and the brightest intellects of your cotemporaries are pursuing with undiminished zeal, the pathway leading to a more complete understanding of the great problems involved in the birth, growth and death of their fellow creatures. So, too, you stand before the gaze of an intelligent public, whose Argus eyes will be quite as likely to discover your foibles and weaknesses as you can well imagine, and by their multiple vision, magnify each indescretion to the extent that you will scarcely be able to recognize the original. In all things, then, let your light so shine before men, that the sharpest professional competition, and the severest public scrutiny may not find you wanting in those qualities of mind and heart, of zeal and steadfastness of purpose, of courage, and a determination to succeed in life, which every one must possess in this age of the world, would he aspire to pre-eminence among his associates, and hold securely the confidence and respect of high minded and intelligent men; and at the same time, assure to himself a reasonable compensation for his personal sacrifices and privations.

What I have said thus far has been for the purpose of inspiring confidence in your own ability, and in the resources of your profession, to meet and overcome all obstacles standing in your pathway, leading to eminence and wealth, while you are elevating the standard of medicine, and benefitting those with whom you associate. Let it not, however, inculcate a spirit of professional pride and bigotry. These are the poorest foundations for preferment, that you could well have—indeed, in most instances, they become positively offensive. They are directly at variance with that respect for your vocation and its duties, and that admiration for those great principles involved in the practice of the science and art of medicine, which I would have the main-spring of your thoughts. Much more might be said upon this subject, but time forbids. It remains for me only to take a more formal leave and bid you farewell. Go hence, remembering that we feel a deep interest in your prosperity or adversity, if fortune shall have such in store, and that we have committed to your keeping, to some extent, the well being of your Alma Mater. May long life and its choicest blessings be yours, and the rich reward of an approving concsience attend the closing acts of your lives.

